# Predictive modeling of bronchopulmonary dysplasia in premature infants: the impact of new diagnostic standards

**DOI:** 10.3389/fped.2024.1434823

**Published:** 2024-10-29

**Authors:** Lijun Tang, Weibin Wu, Weimin Huang, Guangliang Bi

**Affiliations:** Department of Neonatology, Nanfang Hospital, Southern Medical University, Guangzhou, Guangdong, China

**Keywords:** bronchopulmonary dysplasia, prediction model, risk factor, nomogram, premature infant

## Abstract

**Aim:**

To provide a risk prediction for bronchopulmonary dysplasia (BPD) in premature infants under the new diagnostic criteria and establish a prediction model.

**Methods:**

In this study, we retrospectively collected case data on preterm infants admitted to the NICU from August 2015 to August 2018. A lasso analysis was performed to identify the risk factors associated with the development of BPD. A nomogram predictive model was constructed in accordance with the new diagnostic criteria for BPD.

**Result:**

A total of 276 preterm infants were included in the study.The incidence of BPD under the 2018 diagnostic criteria was 11.2%. Mortality was significantly higher in the BPD group than the non-BPD group under the 2018 diagnostic criteria (*P* < 0.05). Fourteen possible variables were selected by the Lasso method, with a penalty coefficient *λ*=0.0154. The factors that eventually entered the logistic regression model included birth weight [BW, OR = 0.9945, 95% CI: 0.9904–0.9979], resuscitation way (OR = 4.8249, 95% CI: 1.3990–19.4752), intrauterine distress (OR = 8.0586, 95% CI: 1.7810–39.5696), score for SNAPPE-II (OR = 1.0880, 95% CI: 1.0210–1.1639), hematocrit (OR = 1.1554, 95% CI: 1.0469–1.2751) and apnea (OR = 7.6916, 95% CI: 1.4180–52.1236). The C-index after adjusting for fitting deviation was 0.894.

**Conclusion:**

This study made a preliminary exploration of the risk model for early prediction of BPD and indicated good discrimination and calibration in premature infants.

## Introduction

In preterm infants, bronchopulmonary dysplasia (BPD) impairs lung development due to various prenatal and postnatal factors (1–3). As the pathophysiology and clinical manifestations of BPD change, perceptions and definitions of the condition evolve over time (4). The widely used criteria for clinical diagnosis and research on BPD in the past was the 2001 National Institute of Child Health and Human Development (NICHD) definition. However, with changes in the characteristics of premature infants surviving in the NICU and ongoing advancements in respiratory support technologies, the 2001 NICHD definition no longer meets the current needs of clinical treatment and research. For example, some infants receiving respiratory support with high flow nasal cannula cannot be classified using the 2001 NICHD definition and may even be misclassified. Additionally, the 2001 NICHD definition categorizes infants at postmenstrual age (PMA) of 36 weeks with FiO_2_ ≥ 30% as severe BPD, regardless of whether they underwent invasive mechanical ventilation, resulting in a wide range in disease severity among these infants and significant differences in clinical outcomes. Importantly, 2001 NICHD definition does not include infants who died early due to respiratory system diseases, even though these infants are more likely to develop BPD or severe BPD.

In 2018, NICHD redefined BPD, incorporatin imaging-confirmed lung consolidation and refining methods of respiratory support, including non-invasive ventilation, now widely accepted in clinical practice. They also categorized oxygen inhalation concentrations and modes of oxygen delivery. Additionally, they highlighted the connection between mechanical ventilation and severe BPD (sBPD) and collected diagnoses of death due to respiratory failure between 14 days and 36 weeks gestational age(GA) to avoid omitting deaths, ensuring epidemiological data integrity of BPD (5).Furthermore, the 2018 NICHD definition have shifted the assessment time frame from postnatal day 28 to PMA36w, reducing false-positive diagnoses under the old criteria and better meeting the needs of clinical practice and research.

The pathogenesis of BPD is highly intricate, and currently, there are no effective treatment measures available (6–8). Therefore, identifying early risk factors and implementing effective measures to reduce BPD severity and incidence is crucial. While BPD has been extensively extensively researched overseas, most studies have utilized the old BPD diagnostic criteria and focused primarily on the European population (9–12). There are distinct variances between the new diagnostic criteria for BPD and the former criteria, which could lead to the identification of novel risk factors. This study aimed to identify relevant risk factors and construct corresponding predictive models based on the new BPD diagnostic criteria through a retrospective analysis, striving for the early identification of high-risk individuals and laying the groundwork for the timely implementation of preventative measures for BPD.

## Methods

### Participants

In order to avoid the potential confounding effects caused by the differences between the old and new BPD diagnostic criteria, we only included cases before 2018 and conducted diagnoses and grouping based on the new BPD diagnostic criteria.The current retrospective cohort study included preterm infants whose GA < 32 weeks in Nanfang Hospital from August 2015 to August 2018. The study excluded individuals meeting the following criteria: ① Patients who discontinued treatment or changed hospitals due to economic reasons. ② Patients who, despite still requiring oxygen at 36 weeks GA, were discharged earlier at their families’ insistence, thereby impeding the collection of follow-up oxygen use data. ③ Patients who passed away between 14 days and 36 weeks GA due to conditions such as necrotizing enterocolitis, severe intraventricular hemorrhage, sepsis, or other causes unrelated to respiratory failure.

### Definitions

According to the 2018 NICHD definition (5) for BPD, preterm infants with GA of <32 weeks at a corrected GA of 36 weeks had imaging evidence of substantial lung disease, and needed different degrees of respiratory support and corresponding levels of FiO_2_ to maintain arterial oxygen saturation between 0.90 and 0.95 for at least 3 consecutive days. Then according to the different respiratory support mode of FiO_2_ into I, II, III level (Table 1).

**Table 1:** NICHD suggested refinements to the definition of BPD.

Grade	Invasive IPPV^a^	N-CPAP, NIPPV, or nasal cannula ≥ 3 L/min	Nasal cannula flow of 1–<3 L/min	Hood O2	Nasal cannula flow of <1 L/min
I	–	21	22–29	22–29	22–70
II	21	22–29	≥30	≥30	>70
III	>21	≥30			
III(A)	Early death (between 14 days of postnatal age and 36 weeks) owing to persistent parenchymal lung disease and respiratory failure that cannot be attributable to other neonatal morbidities (e.g., necrotizing enterocolitis, intraventricular hemorrhage, redirection of care, episodes of sepsis, etc).				

CPAP, continuous positive airway pressure; IPPV, intermittent positive pressure ventilation; N-CPAP, nasal continuous positive airway pressure; NIPPV, noninvasive positive pressure ventilation.

^a^ Excluding infants ventilated for primary airway disease or central respiratory control conditions. Values are percents.

### Clinical data collection

General clinical data included: ① Neonatal characteristics: birth weight (BW), GA, mode of birth (spontaneous delivery, cesarean section), Apgar score at birth, intrauterine distress/asphyxia, resuscitation mode used in the delivery room (respiratory tract cleaning, pressurized oxygen with airbag mask, endotracheal intubation, adrenaline injection, external chest compression); relevant items of scores for neonatal acute physiology-perinatal extension-II (SNAPPE-II). ② Maternal pregnancy data: multiple pregnancy, prenatal use of glucocorticoids, pregnancy-induced diabetes,smoking, ethanol intake and chorioamnionitis; ③ Neonatal complications: neonatal respiratory distress syndrome (NRDS), pulmonary infection, pulmonary hypertension (PH), pulmonary hemorrhage, electrolyte disorder, abnormal coagulation function, hypoproteinemia, hypothyroidism, metabolic bone disease, anemia, shock, cholestasis, sepsis, intracranial infection, intracranial hemorrhage, apnea; ④ Neonatal treatment: aminophylline, caffeine, hydrocortisone, dexamethasone, alveolar surfactant; infusion of various blood products (erythrocyte suspension, plasma, platelets, albumin, gamma globulin); total time of non-invasive auxiliary ventilation, mechanical ventilation, oxygen use; ⑤ Laboratory results: blood routine, coagulation function, procalcitonin, arterial blood gas analysis within 24 h after birth.Intrauterine distress (13), SNAPPE-II (14), pulmonary hypertension (15), apnea (16) were diagnosed based on the specified criteria.

### Data analysis

Statistical Product Service Solutions 20.0 software and R Language Definition 4.0.2 statistical software were used to process all data included in the study. The data were expressed as the mean ± standard deviation (SD), and the comparison between the two groups was done by *t*-test or corrected *t*-test. The data of skew distribution were described by median and interquartile interval (IQR), and the comparison between the two groups was done by the Mann-Whitney *U*-test. The counting data were described by the number of cases and percentage *n* (%). If the frequency was ≥5, Pearson Chi-square test was used; If 1 ≤ frequency <5, the continuity corrected 2 test was used; If the frequency was less than 1, Fisher exact probability method was used. *P* < 0.05 indicated a significance.

## Results

### The incidence and mortality of BPD

For the 276 preterm infants included in the study, BPD was defined based on the 2018 criteria outlined in Table 2. According to these criteria, 31 premature infants were ultimately diagnosed with BPD, resulting in an incidence of 11.2%. Among these cases, there were 17 instances of grade I with no reported mortality, 5 cases of grade II also with no mortality, 7 cases of grade III with a mortality of 14.3%, and 2 cases of grade IIIA with a 100% mortality. The mortality in the BPD group was significantly higher when compared to the non-BPD group (*P* < 0.05). In addition, there were 3 cases of abandonment or death: One case with a GA of 30 ^± 2^ weeks and a BW of 450 g succumbed to respiratory failure after an extended period of treatment. The other two cases, with GA of 26 weeks and 27^+1^ weeks, and BW of 900 g and 1,050 g, respectively, required prolonged mechanical ventilation. Unfortunately, in both cases, the family members eventually opted to discontinue treatment.

**Table 2:** Analysis of morbidity with different BPD classification [*n* (%)].

Diagnostic criteria and graduation	Cases	Abandonment and death
2018 criteria		
BPD group	31 (11.2)	3 (9.7)*
Grade I	17 (6.2)	0 (0)
Grade II	5 (1.8)	0 (0)
Grade III	7 (2.5)	1 (14.3)
Grade IIIA	2 (0.7)	2 (100.0)

BPD, bronchopulmonary dysplasia.

* *P* < 0.05, the mortality compared with non-BPD group in 2018 criteria.

### Comparison of clinical characteristics between two groups

Continuous variables including BW, GA, and 5-min Apgar score, hospital stays, and SNAPPE-II score were compared between groups (Table 3). The results showed that the BPD group exhibited lower BW, GA, and 5-min Apgar score compared to the non-BPD group, but had longer hospital stays and a higher SNAPPE-II score (all *P* < 0.05). Additionally, categorical variables showed that the BPD group had a higher incidence of small for gestational age (SGA), intrauterine distress, endotracheal intubation resuscitation, severe preeclampsia, patent ductus arteriosus (PDA), PH, retinopathy of prematurity (ROP), pulmonary infection, pulmonary hemorrhage, shock, anemia, electrolyte disorder, abnormal coagulation, hypoproteinemia, cholestasis, and apnea compared to the non-BPD group (all *P* < 0.05; Table 3).

**Table 3:** Comparison of clinical characteristics between BPD and non-BPD group.

Items	BPD group (*n* = 31)	Non-BPDgroup (*n* = 245)	*P*
Birth weight (g)	987.10 ± 220.64	1,318.22 ± 259.71	0.000
GA (w)	28.67 ± 1.62	29.71 ± 1.48	0.002
Premature rupture of membranes (>18 h)	6 (19.35)	66 (26.94)	0.365
Length of stay (d)	64[54,73]	38[29,48]	0.000
5-min Apgar (score)	10[9,10]	10[10,10]	0.014
SNAPPE-II (score)	15[10,24]	5[0,7]	0.000
General situation			
Male	19 (61.3)	143 (58.4)	0.848
Cesarean section	17 (54.8)	136 (55.5)	0.944
Multiple pregnancy	9 (29.0)	87 (35.5)	0.476
SGA	7 (22.6)	19 (7.8)	0.016
Intrauterine distress	7 (22.6)	18 (7.3)	0.005
Birth asphyxia	0 (0)	2 (0.8)	1
Corticosteroid use before birth	21 (67.7)	174 (71.0)	0.706
Delivery room resuscitation mode			
Non-intrusive	4 (12.9)	127 (51.8)	0.000
Endotracheal intubation	25 (80.6)	114 (46.5)	
Chest compressions and/or adrenaline	2 (6.5)	4 (1.6)	
Maternal diseases			
Diabetes	2 (6.45)	30 (12.24)	0.515
Preeclampsia	7 (22.6)	16 (6.5)	0.514
Severe preeclampsia	6 (19.4)	13 (5.3)	0.004
Placental abruption	2 (6.5)	11 (4.5)	0.971
Smoking	1 (3.2)	13 5.3）	0.999
Ethanol intake	2 (6.5)	15	0.999
Chorioamnionitis	19	108	0.086
Related complications			
NRDS	30 (96.8)	229 (93.5)	0.745
PDA	8 (25.8)	30 (12.2)	0.039
PH	3 (9.7)	4 (1.6)	0.038
ROP	7 (22.6)	23 (9.4)	0.035
NEC	4 (12.9)	18 (7.3)	0.469
Pulmonary infection	8 (25.8)	9 (3.7)	0.000
Pulmonary hemorrhage	5 (16.1)	4 (1.6)	0.000
Intracranial infection	2 (6.5)	10 (4.1)	0.887
Sepsis	4 (12.9)	20 (8.2)	0.586
Shock	5 (16.1)	9 (3.7)	0.003
Anemia	29 (93.5)	181 (73.9)	0.028
Hypoglycemia	8 (25.8)	37 (15.8)	0.164
Electrolyte disorder	12 (38.7)	23 (9.4)	0.000
Coagulation disorders	14 (45.2)	53 (21.6)	0.007
Hypoproteinemia	18 (58.1)	64 (26.1)	0.000
Cholestasis	10 (32.3)	11 (4.5)	0.000
Apnea			
Incidentally	7 (22.6)	21 (8.6)	0.048
Frequently	2 (6.5)	7 (2.9)	

BPD bronchopulmonary dysplasia; BW, birth weight; GA, gestational age; SGA, small for gestational age; NRDS, neonatal respiratory distress syndrome; PDA, patent ductus arteriosus; PH, pulmonary hypertension; ROP, retinopathy of prematurity; NEC, neonatal necrotizing enterocolitis.

### Comparison of laboratory and therapeutic measures between two groups

The blood results of premature infants within 24 h of birth were used in this study to reflect the most realistic situation of premature infants at birth. In this study, 10 laboratory test indexes including white blood cell count (WBC), neutrophil(NEU), lymphocyte (LYM), hemoglobin (HGB), hematocrit% (HCT%), platelet (PLT), creatinine (Cr), albumin (ALB), procalcitonin (PCT), lactic acid (Lac), prothrombin time (PT), activated partial thromboplastin time (APTT), and fibrinogen (Fib) within 24 h were evaluated. Among them, PLT in the BPD group was significantly lower than that in the non-BPD group, and HCT, Cr and Lac in the BPD group were significantly higher than those in the non-BPD group (all *P* < 0.05; Table 4).

**Table 4:** Comparison of laboratory results and treatment between BPD and non-BPD groups.

Items	Cases	BPD group	Non-BPD group	*P*
Laboratory results				
WBC (×10^9^/L)	274	11.05 ± 5.53	14.09 ± 51.64	0.407
NEU (×10^9^/L)	274	5.91 ± 7.22	5.29 ± 5.02	0.648
LYM (%)	274	47.10 ± 24.42	46.24 ± 18.25	0.815
HGB (g/L)	274	161.07 ± 27.89	154.34 ± 18.85	0.083
HCT (%)	274	0.49 ± 0.07	0.46 ± 0.05	0.017
PLT (×10^9^/L)	274	197.50 ± 72.50	238.75 ± 66.70	0.005
Cr (*μ*mol/L)	247	68.76 ± 45.86	57.27 ± 20.91	0.021
ALB (g/L)	272	28.70 ± 3.86	30.00 ± 3.87	0.088
PCT (ng/ml)	231	10.59 ± 22.70	14.06 ± 24.75	0.505
Lac (mmol/L)	270	4.21 ± 2.59	3.15 ± 2.36	0.037
PT (s)	126	20.58 ± 4.54	21.01 ± 22.17	0.860
APTT (s)	126	102.05 ± 37.17	93.25 ± 36.47	0.373
Fib (g/L)	126	1.64 ± 1.29	1.95 ± 1.23	0.362
D-dimer (mg/L FEU)	116	17.16 ± 27.94	9.22 ± 19.32	0.291
Respiratory therapy				
Initial FiO_2_ (%) within first day after birth	276	29.81 ± 9.134	26.09 ± 10.29	0.056
Maximum FiO_2_ (%) within 7 days after birth	276	31.77 ± 10.01	28.74 ± 11.48	0.162
Non-positive pressure ventilation time (d)	276	19.07 ± 11.57	9.28 ± 8.29	0.000
Non-invasive auxiliary ventilation time (d)	276	26.93 ± 16.60	10.97 ± 10.68	0.000
Mechanical ventilation time (d)	276	15.09 ± 18.87	2.48 ± 5.03	0.000
Total oxygen consumption time (d)	276	61.08 ± 17.19	22.73 ± 17.18	0.000
Mechanical ventilation time within 7 days after birth (d)	276	4.60 ± 3.02	1.54 ± 2.28	0.000
Early preventive drugs				
Aminophylline	276	13 (45.2)	165 (67.3)	0.044
Caffeine	276	3 (9.7)	16 (7.3)	
PS (times)	276	1.52 ± 0.93	1.10 ± 0.71	0.003
PS types				
Kelisu	276	16	153	0.136
Curosurf	276	11	55	
Kelisu and curosurf	276	2	4	
Hydrocortisone	276	16 (51.6)	15 (6.1)	0.000
Dexamethasone	276	20 (64.5)	52 (21.2)	0.000
Blood products				
Erythrocyte suspension^a^	276	6.45 ± 3.31	2.41 ± 2.30	0.000
Erythrocyte suspension	276	1.08 ± 1.15	0.50 ± 0.97	0.025
Plasma^a^	276	1.67 ± 1.51	2.08 ± 1.51	0.417
Plasma	276	0.31 ± 0.66	0.18 ± 0.76	0.313
PLT^a^	276	0	1.00 ± 1.00	0.089
PLT	276	0.14 ± 0.58	0.03 ± 0.24	0.067
Gamma globulin^a^	276	1.45 ± 1.47	1.11 ± 1.20	0.367
Gamma globulin	276	0.47 ± 0.64	0.70 ± 0.69	0.248
ALB^a^	276	3.36 ± 8.33	0.33 ± 0.59	0.033
ALB	276	0.24 ± 0.51	0.11 ± 0.38	0.101

BPD, bronchopulmonary dysplasia; WBC, white blood cell; NEU, neutrophil; LYM, lymphocyte; HGB, hemoglobin; HCT, hematocrit; PLT, platelets; Cr, creatinine; ALB, albumin; PCT, procalcitonin; Lac, lactic acid; PT, prothrombin time; APTT, activated partial thromboplastin time; Fib, fibrinogen; PS, pulmonary surfactant.

^a^ indicates the use of blood products in premature infants within 7 days after birth.

The oxygen therapy was compared between two groups (Table 4). In the BPD group, the total time of oxygen use, non-positive pressure ventilation, non-invasive auxiliary ventilation, and the duration of mechanical ventilation were increased compared to the non-BPD group (all *P* < 0.05).

Drug administration between the two study groups was meticulously assessed and the findings were detailed in Table 4. Notably, a comparison revealed a statistically significant increase in the usage of pulmonary surfactants (PS) such as Kelisu, Curosurf, and their combination, as well as hydrocortisone and dexamethasone in the BPD group compared to the non-BPD group (*P* < 0.05). Additionally, it was observed that the incidence of BPD in the aminophylline-treated group was notably lower than in the non-aminophylline-treated group (*P* < 0.05). Nonetheless, the analysis did not uncover any significant correlation between the administration of various types of PS and the development of BPD (*P* > 0.05).

Furthermore, the administration of blood products including erythrocyte suspension, plasma, platelets (PLT), gamma globulin, and albumin was rigorously evaluated in both study groups. Table 4 illustrated that the infusion frequency of erythrocyte suspension was significantly higher in the BPD group compared to the non-BPD group (*P* < 0.05). However, there were no discernible differences in the utilization of other blood products between the groups (*P* > 0.05). Moreover, a targeted examination focusing specifically on premature infants aged 7 days was undertaken. The results revealed a marked increase in the transfusions of erythrocyte suspension and albumin in premature infants within the BPD group in comparison to the non-BPD group (*P* < 0.05). Noteworthy, there were no significant distinctions in the utilization rates of plasma, platelets, and gamma globulin between the study groups (*P* > 0.05).

### Establishment of an early risk prediction model for BPD

**Figure 1:**
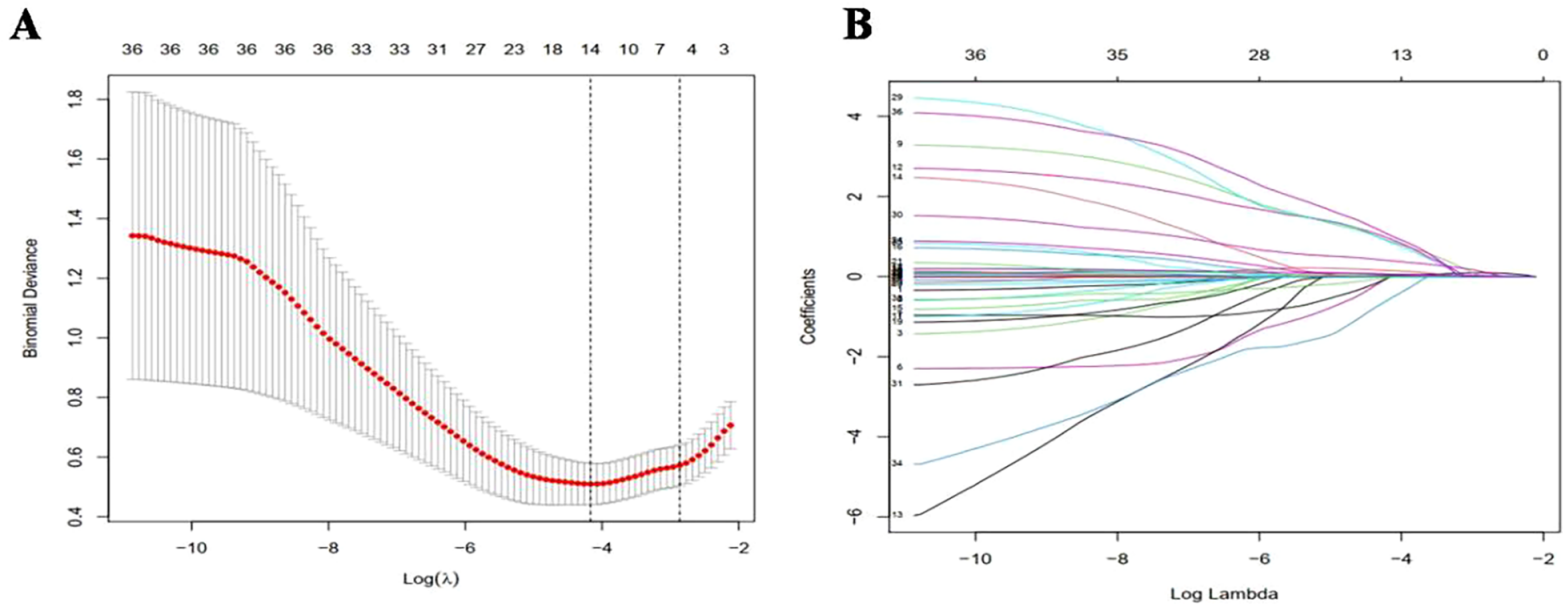
Variable selection and penalty coefficient determination in lasso regression model. **(A)** In Lasso model, the best penalty coefficient was selected by 10 fold alternating verification method and minimization standard. The average binomial deviation and logarithm were plotted. Two dotted lines on the left and right were the minimum log(*λ*) and maximum log(*λ*) within 1 times standard error, respectively. **(B)** Penalty diagram of 36 variable coefficients. With the changes of penalty coefficient *λ*, the variable system was compressed, and then most of the variable coefficients were compressed to 0. At the minimum log(*λ*), 14 variables with non-zero coefficients were selected.

The study outcomes demonstrated a clear distinction between patients with BPD and those without BPD, as shown in Figure 1. This study involved a large number of variables, and there may also be linear or causal relationships between variables. In order to eliminate the problem of overfitting in regression models caused by these situations, the study first used Lasso regression for analysis. Utilizing the lasso method for variable screening, the optimal penalty coefficient *λ* for the model was determined to be 0.0154 through cross-validation and the original 36 variables were reduced to 14 possible risk factors: mechanical ventilation time, early preventive drugs, BW, resuscitation mode, 5 min-Apgar score, intrauterine distress, SNAPPE-II score, neonatal necrotizing enterocolitis (NEC), creatinine (Cr), early use of hydrocortisone, frequency of ALB, HCT, frequency of PLT and apnea,as shown in Figure 1.

Regression coefficient, odds ratio (OR) value, 95% confidence interval (CI), and *p*-value are presented in Table 5. The results revealed that only BW, resuscitation mode, intrauterine distress, SNAPPE-II score, HCT, and apnea were significant factors included in the regression equation (*p* < 0.05). The corresponding logistic regression equation is as follows: Z = −5.7524–0.0055 × BW + 1.5738 × Recovery mode + 2.0867 × Intrauterine distress + 0.0844 × SNAPPE-II + 0.1444 × HCT + 2.0401 × Apnea.

**Table 5:** Early prediction of risk factors for BPD.

Intercept and variable	Prediction model		
	*β*	OR value (95%CI)	*P*
Intercept	−5.7524	0.0032（9.90 × 10^−7^–6.4360）	0.0006
BW	−0.0055	0.9945（0.9904–0.9979）	0.0037
Recovery mode	1.5738	4.8249（1.3990–19.4752）	0.0178
Intrauterine distress	2.0867	8.0586（1.7810–39.5696）	0.0073
SNAPPE-II	0.0844	1.0880（1.0210–1.1639）	0.0101
HCT	0.1444	1.1554（1.0529–1.2847）	0.0039
Apnea	2.0401	7.6916（1.4180–52.1236）	0.0258

BPD, bronchopulmonary dysplasia; BW, birth weight; HCT, hemotocrit; OR, odds ratio; CI, confidence interval.

β is the regression coefficient.

To facilitate the clinical application of these results, we developed a nomogram that incorporates the six statistically significant variables. This tool serves as a user-friendly interface for rapidly estimating the risk factors for BPD in preterm infants on the 7th day of hospitalization. The input variables of the nomogram directly correspond to the aforementioned six predictors, and its output consists of individual score scales for each input, a cumulative score, as well as the associated risk probability of BPD occurrence.

**Figure 2:**
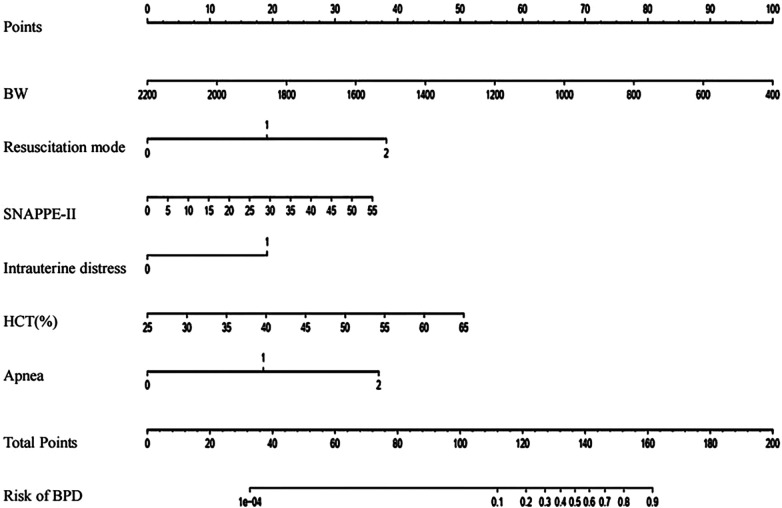
Prediction nomogram for BPD risk. To utilize this nomogram, begin by locating the particular value for each patient on the respective variable axis. Drawing a line upwards from this point will determine the corresponding points for each variable. Afterwards, add up the total points and identify the same score on the "Total Points axis". A vertical line drawn downwards from this point will indicate the probability of BPD. BW, birth weigh; HCT, hematocrit.

Illustratively, Figure 2 demonstrated the functionality of the nomogram in a hypothetical clinical scenario. Consider a preterm infant with a birth weight of 1000g, who underwent resuscitation via endotracheal intubation in the delivery room, exhibited intrauterine distress, and presented with a HCT of 0.450, a SNAPPE-II score of 25 within 24 h of admission, and occasional apnea seven days post-admission. For this infant, the nomogram assigns individual scores of 67, 19, 19, 25, 17, and 18 points, respectively, to the six predictors. The cumulative score of 165 translates to a BPD risk exceeding 90%, thereby underscoring the high likelihood of BPD in this particular infant. This personalized risk assessment underscores the clinical utility of our predictive model in identifying preterm infants at increased risk for BPD.

### Distinction and calibration of model

**Figure 3:**
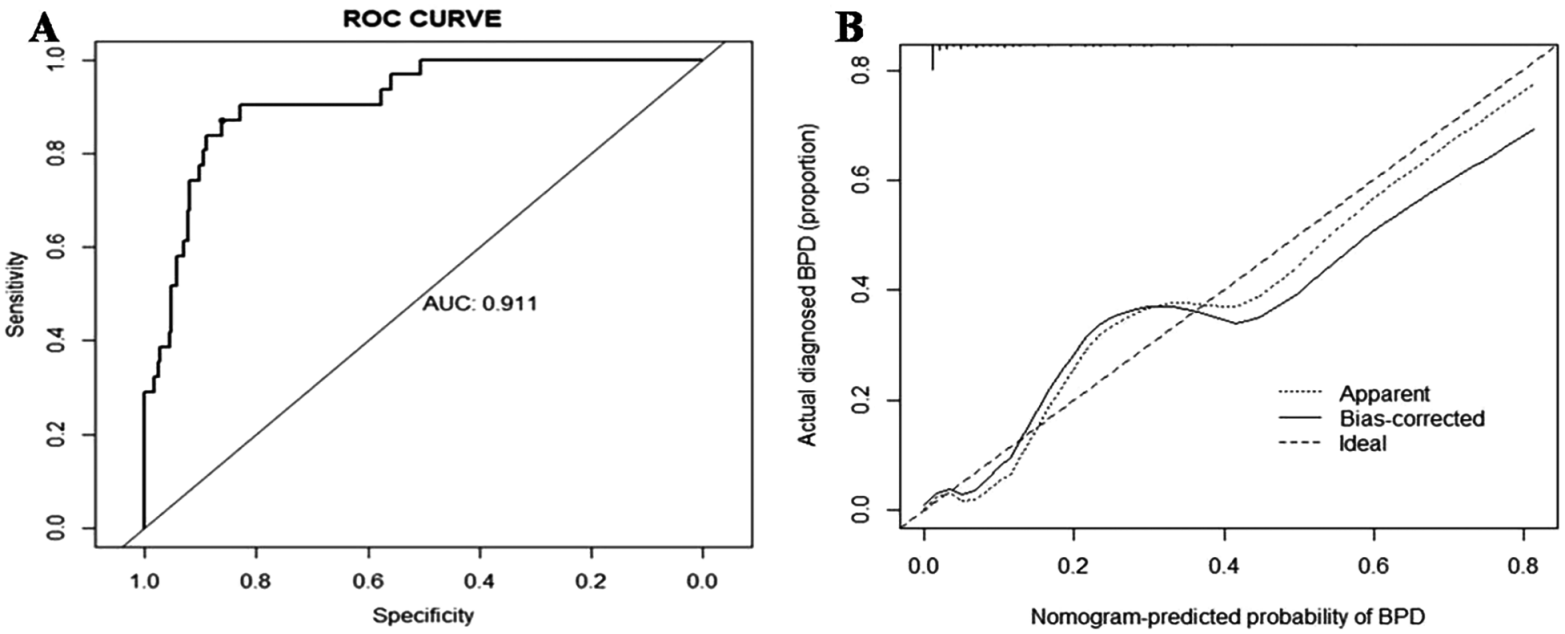
The ROC and calibration curves of the nomogram for BPD. **(A)** The ROC curve of BPD events in premature infants. **(B)** The calibration curve of BPD events in premature infants. ROC, receiver operating characteristic; BPD, bronchopulmonary dysplasia.

For the regression model described above, it was necessary to evaluate its discrimination and calibration. The degree of discrimination was calculated by the AUC value through the Receiver Operating Characteristic(ROC) curve (c-index). As shown in Figure 3, the c-index  was found to be 0.911 (95% CI: 0.862–0.960). The bootstrap method was used for 1,000 resampling internal verification, and c-index = 0.894 was calculated after correcting for fitting bias. This finding indicated that the risk factor prediction model possesses robust discrimination ability. The degree of calibration was evaluated using the calibration curve, as shown in Figure 3. The dotted line approximately represents the distribution curve of the apparent predicted value of the logistic regression model in this study, while the bias-corrected curve represents the distribution curve after adjusting for bias. The ideal curve represents the perfect distribution. The results demonstrated a high degree of the coincidence between the approximate curve and the bias-corrected curve, both aligning well with the ideal curve. The Hosmer-Lemeshow goodness-of -fit test yielded a *P*  -value of 0.90, indicating excellent calibration of the model.

## Discussion

In our study, 276 premature infants aged 25 to 31^+6^ weeks were diagnosed with BPD according to the 2018 definition, with an incidence of 11.2%. The incidence of BPD in China was generally lower compared to other countries (17), mainly attributed to the higher medical expertise abroad, which allowed for the successful treatment of even younger and lighter premature infants, leading to higher BPD rates. The more detailed and stringent BPD criteria in the 2018 definition also played a significant role.Under the 2001 NICHD definition, patients with mild BPD may not have met the diagnostic criteria for BPD according to the 2018 NICHD definition, resulting in a noticeable reduction in the incidence. This optimization helped conserve medical resources by focusing on patients with severe BPD, enhancing the allocation of resources effectively.

In this investigation, variable selection was conducted based on a thorough review of pertinent clinical literature and enabled by access to comprehensive data on hospitalized newborns spanning the prenatal to postnatal periods (18, 19). Single factor analysis revealed statistically significant differences between groups for key variables such as BW, GA, 5-minute Apgar score, SNAPPE-II score, SGA, presence of intrauterine distress, method of recovery, severe preeclampsia, PDA, duration of ventilator support, use of PS, and frequency of red cell transfusions. Consistent with findings from both domestic and international studies, risk factors for BPD are closely linked to fetal age, weight, duration of respiratory support, presence of PDA, and episodes of asphyxia (12, 20–24). These critical insights underscore the importance of vigilance towards factors that may contribute to the development of BPD in the clinical management of premature infants.

Our study utilized the logistic regression method to establish a risk prediction model, which primarily considered six variables: BW, recovery mode, intrauterine distress, SNAPPE-II score, HCT, and apnea incidence. The Lasso method was employed to mitigate overfitting of the regression models. Our study also evaluated the predictive efficacy of the model, demonstrating its strong ability to distinguish BPD risk. This research represents a preliminary exploration of an early risk prediction model for BPD, providing valuable insights for clinical management of premature infants. Heightened vigilance and early intervention may be warranted for infants with high predicted BPD risk.

Below we specifically discuss the six risk factors enrolled in the regression model.The most important risk factor for the occurrence of BPD is premature birth and low BW (25). A multicenter survey conducted in China reported a high incidence of BPD in extremely low BW infants in 2019 (26). Research conducted abroad shows that 95% of patients with BPD are born with extremely low BW (27). Additionally, BW is an important indicator for early prediction of BPD (28). In our study of 31 patients with BPD, 20 cases (64.5%) were born with extremely low BW, and 11 cases (35.5%) were born with very low BW. Of the 31 patients with BPD, 11 (35.5%) had a GA less than 28 weeks, and 10 (32.3%) had a GA between 28 and 30 weeks. We considered entering BW instead of GA into the regression equation because low BW implies poor development, and a higher GA with low BW (i.e., lower than gestation) also implies poor development. Therefore, BW is a more accurate predictor than GA.

We also found that intrauterine fetal distress entered the regression model, indicating that fetuses with intrauterine distress are more likely to develop BPD. This may be because intrauterine distress can cause fetal hypoxia, leading to low Apgar scores, and increased need for tracheal intubation. Low Apgar scores and tracheal intubation are closely related to the occurrence of BPD (29). Our regression equation also included the method of delivery room resuscitation. Infants who require early tracheal intubation or even the use of adrenaline or chest compressions for resuscitation have poorer lung development and birth conditions, making them more susceptible to BPD. During clinical diagnosis and treatment, it is recommended to establish and maintain functional residual capacity through interventions such as the “T-piece resuscitator” and Continuous Positive Airway Pressure (CPAP) for premature infants with spontaneous respiration, in order to avoid tracheal intubation and mechanical ventilation and reduce the incidence of BPD (30). Additionally, we found that respiratory pauses are associated with the occurrence of BPD. Repeated respiratory pauses can cause tissue hypoxia in the lungs, affecting the development of alveoli and pulmonary vessels.

One innovative aspect of this study is the introduction of a new method to predict BPD, called the Score for Neonatal Acute Physiology-II and Perinatal Extension (SNAPPE-II). This is a scoring system used for early prediction of neonatal mortality risk by evaluating the physiology of the infant and perinatal conditions within 24 h of admission (31, 32). The score includes 9 scoring items, including mean arterial pressure, lowest body temperature, ratio of arterial oxygen pressure to inspired oxygen concentration, lowest blood pH, repeated convulsions, urine output, body weight, being SGA, and Apgar score < 7 at 5 min. Each scoring item is assigned different weight values, with higher scores indicating more severe illness and higher risk of mortality. In our study, the SNAPPE-II score for the BPD group was significantly higher than that for the non-BPD group. The AUC value of SNAPPE-II for predicting the occurrence of BPD was 0.792 (95% CI: 0.707–0.876), with a cutoff value of 11, and sensitivity, specificity, and Youden's index of 80.6%, 76.0%, and 56.6%, respectively. The results showed that SNAPPE-II has a good predictive ability for BPD, and a score higher than 11 indicates a high risk of BPD occurrence. The risk of BPD occurrence is related to the GA and BW of premature infants, as indicated by the “birth weight” and “small for gestational age” items in SNAPPE-II, which are the items with higher scores in this study. The item “ratio of arterial oxygen pressure to inspired oxygen concentration” represents the early oxygenation capacity of premature infants, with better lung function in premature infants being able to maintain good arterial oxygen pressure at a lower inspired oxygen concentration. SNAPPE-II also entered the early prediction regression equation for BPD. Therefore, in clinical practice, it is feasible and effective to use SNAPPE-II either alone or in combination with other risk factors to early predict the occurrence of BPD.

In this study, we also found an interesting result: HCT is an indicator for early prediction of BPD. Previous studies have found that the level of HCT < 0.455 at 1 week after birth was also an independent risk factor for BPD, as suggested by multivariate logistic regression (33). This could be explained as follows: the HCT test measures the proportion of red blood cells, which carry oxygen throughout the body, and the HCT value determines the oxygen-carrying capacity of the blood. Therefore, preterm infants with low HCT levels from birth to the first week of life may have inadequate oxygen and nutrient supply, which may adversely impact lung growth and development (34). A meta-analysis found that anemia requiring blood transfusion is closely related to the occurrence of BPD (35). The reason for this analysis may be that the transfused blood has lower adult hemoglobin oxygen affinity, and transfusion increases free oxygen content in tissues, promoting the occurrence of cell oxygen toxicity and BPD (10).

The diagnosis of BPD based on both old and new criteria often occurs relatively late, namely at postnatal day 28 or postmenstrual age 36 weeks, which is unfavorable for the prevention and treatment of BPD in clinical practice. Therefore, the identification of high-risk factors and early prediction has become a hotspot in clinical research. However, current studies mostly adhere to the old diagnostic criteria, lacking analyses of the influencing factors and the establishment of prediction models based on the 2018 new diagnostic criteria.This study explored the high-risk factors for BPD before birth and during the first week after birth under the new diagnostic criteria from the NICHD. By establishing an early risk prediction model at postnatal week one, this study effectively identified infants born preterm at less than 32 weeks who are at high risk of developing BPD. This provides clinicians with an accurate and effective tool for predicting and preventing BPD, enabling personalized interventions at an early stage.In contrast to many previous studies, this research incorporated critical illness scoring commonly used in clinical practice to assess the severity of newborn conditions, in conjunction with other high-risk factors. This approach enhanced the screening process for identifying high-risk infants for BPD, offering valuable support to clinicians in their decision-making processes.

Our study also has certain limitations. First, this is a retrospective single-center study, which may have some degree of selection bias and may not be representative of all children with BPD. Moreover, BPD incidence rates vary across different regions and hospitals. Additionally, although we have collected the relevant clinical data, due to limitations, we were unable to include related biomarkers and other high-risk factors for BPD, such as the mode of mechanical ventilation and placental pathology results. We hope to conduct a prospective, multicenter study in the future, establishing different assessment points during hospitalization (such as on the 1st, 7th, and 14th days after birth). This future study would also incorporate relevant biomarkers and include post-discharge follow-ups to track readmission rates and the incidence of respiratory diseases. The aim would be to build a comprehensive and accurate predictive model. In this study, risk models for early prediction of BPD were explored, indicating BW, recovery mode, intrauterine distress, SNAPPE-II score, HCT, and apnea as risk factors for early prediction of BPD in premature infants <32 weeks. However, conducted at a single center with inadequate cases, future studies should be multi-center with follow-up at different time points to establish a comprehensive, optimal prediction model.

## Data Availability

The raw data supporting the conclusions of this article will be made available by the authors, without undue reservation.

## References

[1] Thebaud B Goss KN Laughon M Whitsett JA Abman SH Steinhorn RH Bronchopulmonary dysplasia. Nat Rev Dis Primers. (2019) 5(1):78. 10.1038/s41572-019-0127-7 31727986 PMC6986462

[2] Hwang JS Rehan VK. Recent advances in bronchopulmonary dysplasia: pathophysiology, prevention, and treatment. Lung. (2018) 196(2):129–38. 10.1007/s00408-018-0084-z 29374791 PMC5856637

[3] Gilfillan M Bhandari A Bhandari V. Diagnosis and management of bronchopulmonary dysplasia. Br Med J. (2021) 375:n1974. 10.1136/bmj.n1974 34670756

[4] Cuevas GM Dahm PH Welty SE. The challenge of accurately describing the epidemiology of bronchopulmonary dysplasia (BPD) based on the various current definitions of BPD. Pediatr Pulmonol. (2021) 56(11):3527–32. 10.1002/ppul.25434 33913625

[5] Higgins RD Jobe AH Koso-Thomas M Bancalari E Viscardi RM Hartert TV Bronchopulmonary dysplasia: executive summary of a workshop. J Pediatr. (2018) 197:300–8. 10.1016/j.jpeds.2018.01.043 29551318 PMC5970962

[6] Jensen EA Roberts RS Schmidt B. Drugs to prevent bronchopulmonary dysplasia: effect of baseline risk on the number needed to treat. J Pediatr. (2020) 222:244–7. 10.1016/j.jpeds.2020.01.070 32143932

[7] Harris C Greenough A. The prevention and management strategies for neonatal chronic lung disease. Expert Rev Respir Med. (2023) 17(2):143–54. 10.1080/17476348.2023.2183842 36813477

[8] Abiramalatha T Ramaswamy VV Bandyopadhyay T Somanath SH Shaik NB Pullattayil AK Interventions to prevent bronchopulmonary dysplasia in preterm neonates: an umbrella review of systematic reviews and meta-analyses. JAMA Pediatr. (2022) 176(5):502–16. 10.1001/jamapediatrics.2021.6619 35226067

[9] Shim SY Yun JY Cho SJ Kim MH Park EA. The prediction of bronchopulmonary dysplasia in very low birth weight infants through clinical indicators within 1 hour of delivery. J Korean Med Sci. (2021) 36(11):e81. 10.3346/jkms.2021.36.e81 33754511 PMC7985290

[10] Sharma A Xin Y Chen X Sood BG. Early prediction of moderate to severe bronchopulmonary dysplasia in extremely premature infants. Pediatr Neonatol. (2020) 61(3):290–9. 10.1016/j.pedneo.2019.12.001 32217025

[11] Jassem-Bobowicz JM Klasa-Mazurkiewicz D Zawrocki A Stefanska K Domzalska-Popadiuk I Kwiatkowski S Prediction model for bronchopulmonary dysplasia in preterm newborns. Children (Basel). (2021) 8(10):886. 10.3390/children8100886 34682151 PMC8534367

[12] Romijn M Dhiman P Finken M van Kaam AH Katz TA Rotteveel J Prediction models for bronchopulmonary dysplasia in preterm infants: a systematic review and meta-analysis. J Pediatr. (2023) 258:113370. 10.1016/j.jpeds.2023.01.024 37059387

[13] The ACOG Committee. ACOG practice bulletin no. 106: intrapartum fetal heart rate monitoring: nomenclature, interpretation, and general management principles. Obstet Gynecol. (2009) 114(1):192–202. 10.1097/AOG.0b013e3181aef106 19546798

[14] Richardson DK Corcoran JD Escobar GJ Lee SK. SNAP-II and SNAPPE-II: simplified newborn illness severity and mortality risk scores. J Pediatr. (2001) 138(1):92–100. 10.1067/mpd.2001.109608 11148519

[15] Clark RH Kueser TJ Walker MW Southgate WM Huckaby JL Perez JA Low-dose nitric oxide therapy for persistent pulmonary hypertension of the newborn. Clinical inhaled nitric oxide research group. N Engl J Med. (2000) 342(7):469–74. 10.1056/NEJM200002173420704 10675427

[16] Eichenwald EC. Apnea of prematurity. Pediatrics. (2016) 137(1):e20153757. 10.1542/peds.2015-3757 26628729

[17] Baud O Laughon M Lehert P. Survival without bronchopulmonary dysplasia of extremely preterm infants: a predictive model at birth. Neonatology. (2021) 118(4):385–93. 10.1159/000515898 34004607

[18] Muehlbacher T Bassler D Bryant MB. Evidence for the management of bronchopulmonary dysplasia in very preterm infants. Children (Basel). (2021) 8(4):298. 10.3390/children8040298 33924638 PMC8069828

[19] Philpot PA Bhandari V. Predicting the likelihood of bronchopulmonary dysplasia in premature neonates. Expert Rev Respir Med. (2019) 13(9):871–84. 10.1080/17476348.2019.1648215 31340666

[20] Poets CF Lorenz L. Prevention of bronchopulmonary dysplasia in extremely low gestational age neonates: current evidence. Arch Dis Child Fetal Neonatal Ed. (2018) 103(3):F285–91. 10.1136/archdischild-2017-314264 29363502

[21] Yang T Shen Q Wang S Dong T Liang L Xu F Risk factors that affect the degree of bronchopulmonary dysplasia in very preterm infants: a 5-year retrospective study. BMC Pediatr. (2022) 22(1):200. 10.1186/s12887-022-03273-7 35413820 PMC9004103

[22] Alvarez-Fuente M Moreno L Mitchell JA Reiss IK Lopez P Elorza D Preventing bronchopulmonary dysplasia: new tools for an old challenge. Pediatr Res. (2019) 85(4):432–41. 10.1038/s41390-018-0228-0 30464331

[23] Gao Y Liu D Guo Y Cao M. Risk prediction of bronchopulmonary dysplasia in preterm infants by the nomogram model. Front Pediatr. (2023) 11:1117142. 10.3389/fped.2023.1117142 36999082 PMC10043170

[24] Dai SZ Li SS Zhou MY Xu Y Zhang L Zhang YH Assessment of risk factors for bronchopulmonary dysplasia with pulmonary hypertension and construction of a prediction nomogram model. Zhonghua Er Ke Za Zhi. (2023) 61(10):902–9. 10.3760/cma.j.cn112140-20230616-00406 37803857

[25] Stoll BJ Hansen NI Bell EF Walsh MC Carlo WA Shankaran S Trends in care practices, morbidity, and mortality of extremely preterm neonates, 1993–2012. JAMA. (2015) 314(10):1039–51. 10.1001/jama.2015.10244 26348753 PMC4787615

[26] Collaborative Study Group for Extremely Preterm and Extremely Low Birth Weight Infants. Short-term outcomes and their related risk factors of extremely preterm and extremely low birth weight infants in Guangdong province. Zhonghua Er Ke Za Zhi. (2019) 57(12):934–42. 10.3760/cma.j.issn.0578-1310.2019.12.008 31795560

[27] Walsh MC Yao Q Gettner P Hale E Collins M Hensman A Impact of a physiologic definition on bronchopulmonary dysplasia rates. Pediatrics. (2004) 114(5):1305–11. 10.1542/peds.2004-0204 15520112

[28] Sucasas-Alonso A Pertega-Diaz S Balboa-Barreiro V Garcia-Munoz RF Avila-Alvarez A. Prediction of bronchopulmonary dysplasia in very preterm infants: competitive risk model nomogram. Front Pediatr. (2024) 12:1335891. 10.3389/fped.2024.1335891 38445078 PMC10912561

[29] Yang Y He X Zhang X Chen P. Clinical characteristics of bronchopulmonary dysplasia in very preterm infants. Zhong Nan Da Xue Xue Bao Yi Xue Ban. (2023) 48(10):1592–601. 10.11817/j.issn.1672-7347.2023.230192 38432888 PMC10929901

[30] Sweet DG Carnielli VP Greisen G Hallman M Klebermass-Schrehof K Ozek E European consensus guidelines on the management of respiratory distress syndrome: 2022 update. Neonatology. (2023) 120(1):3–23. 10.1159/000528914 36863329 PMC10064400

[31] Chen CY Huang WM Qian XH Tang LJ. A comparative analysis of neonatal critical illness score and score for neonatal acute physiology, perinatal extension, version II. Zhongguo Dang Dai Er Ke Za Zhi. (2017) 19(3):342–5. 10.7499/j.issn.1008-8830.2017.03.018 28302209 PMC7390156

[32] Dammann O Naples M Bednarek F Shah B Kuban KC O’Shea TM SNAP-II and SNAPPE-II and the risk of structural and functional brain disorders in extremely low gestational age newborns: the ELGAN study. Neonatology. (2010) 97(2):71–82. 10.1159/000232588 19672122 PMC2790760

[33] Wang X Wang S Chen M Lv Y Chen X Yang C. The value of hematocrit for predicting bronchopulmonary dysplasia in very low birth weight preterm infants. Medicine (Baltimore). (2023) 102(39):e35056. 10.1097/MD.0000000000035056 37773858 PMC10545317

[34] Pereira DJS den Dekker HT de Jongste JC Reiss IK Steegers EA Jaddoe V Maternal hemoglobin and hematocrit levels during pregnancy and childhood lung function and asthma. The generation R study. Pediatr Pulmonol. (2018) 53(2):130–7. 10.1002/ppul.23733 29265553

[35] Tang L Zhu TT Zhao J. Association between red blood cell transfusion and bronchopulmonary dysplasia: a systematic review and meta-analysis. Front Pediatr. (2023) 11:1095889. 10.3389/fped.2023.1095889 37325359 PMC10266411

